# Exploring unmet healthcare needs and associated inequalities among middle-aged and older adults in Eastern China during the progression toward universal health coverage

**DOI:** 10.1186/s13561-024-00521-7

**Published:** 2024-06-27

**Authors:** Yunhan Wang, Nan Jiang, Haiya Shao, Zhonghua Wang

**Affiliations:** 1 School of Health Policy & Management, Nanjing Medical University, Nanjing, 211166 China; 2 The Public Health Policy and Management Innovation Research Team, Nanjing Medical University, Nanjing, 211166 China; 3 School of Public Health, Nanjing Medical University, Nanjing, 211166 China

**Keywords:** Universal health coverage, Unmet healthcare needs, Inequality, Concentration index, China

## Abstract

**Background:**

Given the rapid population aging in China, achieving universal health coverage (UHC) presents a primary challenge in addressing unmet healthcare needs and associated inequalities among middle-aged and older adults. Several studies have focused on healthcare utilization and its inequalities, but little attention has been paid to the inequality in unmet healthcare needs. This study aimed to analyze the inequalities in unmet the healthcare needs of middle-aged and older adults in eastern China during the progression toward UHC.

**Methods:**

Data were obtained from the fourth, fifth, and sixth National Health Service Survey (NHSS) of Jiangsu Province, located in eastern China, during the years 2008, 2013, and 2018, respectively. Logistic regression models were used to assess the associated factors of unmet healthcare needs. The inequality was measured according to the concentration index (CI) and its decomposition.

**Results:**

In this study, we found that 12.86%, 2.22%, and 48.89% of middle-aged and older adults reported unmet needs for outpatient and inpatient services and physical examinations, respectively. The prevalence of unmet outpatient needs increased from 2008 to 2018, while the prevalence of unmet inpatient services was lower but maintained. The prevalence of unmet needs for physical examinations among middle-aged and older adults markedly decreased since 2008. Rural areas had a higher prevalence of unmet needs for inpatient services and physical examinations than urban areas. Unmet healthcare needs were more prevalent among the poor. The pro-poor inequalities of unmet healthcare needs have been mitigated during the progression toward UHC; however, they remain predominant among rural middle-aged and older adults for outpatient and inpatient services. Socioeconomic factors significantly influenced unmet healthcare needs and contributed to their inequalities.

**Conclusions:**

The findings characterize the prevalence and inequality of unmet healthcare need among middle-aged and older adults in eastern China during the progression toward UHC. Policy interventions should be actively advocated to effectively mitigate the unmet healthcare needs and address the associated inequalities.

## Background

Universal health coverage (UHC) is a system that provides all citizens of a country or region with access to quality health services without being denied treatment due to their income, social status, or any other factors. It encompasses a broad range of preventive, curative, and palliative care services, including primary healthcare, maternity care, mental health services, and emergency care, among others [1, 2]. Attaining UHC is one of the Sustainable Development Goals (SDGs) established by the international community for 2030, and it has been a key objective of the Healthy China Initiative since 2017.

A fundamental challenge in achieving UHC is healthcare inequality, which has become a major priority for health systems globally [3]. Healthcare inequality refers to the unnecessary, avoidable, and unfair disparities in healthcare access caused by socially determined factors such as income, education, gender, and geographic location. Some individuals or groups have less access to healthcare services than others, despite similar health needs or conditions. To mitigate healthcare inequality and provide quality, affordable, and equitable healthcare for all citizens, China introduced the New Health Care Reform (NHCR) in 2009. This reform includes expanding health insurance coverage and improving the health system. As of now, a preliminary framework for a basic medical insurance system in China has been established; the number of people participating in basic medical insurance has exceeded 1.3 billion, with a coverage rate of more than 95% [4], indicating that China is moving toward universal health coverage. Specially, in Jiangsu Province, the coverage rate of basic medical insurance showed an increasing trend from 2008 to 2018, reaching over 97% by 2018, nearly achieving universal coverage for the population [5]. This improvement contributed to a reduction in the incidence of catastrophic health expenditure in the province [6]. However, healthcare utilization in China remains highly unequal across different subpopulations or regions. Understanding how well a health system meets the needs of the population is critical to achieving the policy aspirations of UHC [7]. As an indicator of lack of access, unmet healthcare needs are defined as subjective perceptions of not receiving appropriate health services when required [8]. In contrast to healthcare utilization, which solely reflects the actual use of health services, unmet healthcare needs reveal the gap between the perceived need for healthcare and actual utilization and serve as a reliable indicator for accurately assessing the accessibility of healthcare [9]. Unmet healthcare need is one of the most important issues of access to healthcare that need to be addressed, as it can lead to poorer health outcomes in the population, increase health and healthcare inequalities, and affect UHC [10, 11].

Middle-aged and older adults refer to individuals who are typically aged 45 and above. According to the United Nations Population Division, there were approximately 78.5 million adults aged 65 years or older in 2021, accounting for 10.2% of the global population. By 2050, this number is projected to almost triple, reaching 21.5 billion to represent one-third of the population. China’s aging problem is even worse, with its older adult population aged 60 years or older accounting for 18.9% of the total population as of the end of 2021. However, it is expected to exceed 30% by 2035. Older adults are more sensitive to healthcare utilization because of the high prevalence of chronic disease, low incomes, and the absence of social security. Several studies have targeted healthcare utilization and its inequality among middle-aged and older adults. Vahedi et al. reported that the primary factors contributing to the increased inequality in the utilization of outpatient and inpatient services in Iran are residence in rural areas and supplementary insurance [12]. Johar et al. reported that a pro-poor inequality in outpatient services utilization exists while access to most other forms of healthcare is best for the rich [13]. Somkotra identified a pro-rich inequality in dental care utilization, which persisted in the older adult population after the country achieved universal coverage [14]. Joe et al. reported that actual healthcare utilization is concentrated among the affluent, and income plays a role in pro-rich inequality [15]. Several studies have explored unmet healthcare needs among middle-aged and older adults. Mielck et al. found that older people with poor self-reported health status and lower income were more likely to report unmet healthcare needs [16]. Eimontas et al. suggested that unmet healthcare needs negatively affect mental health in older adults [17]. Momtaz et al. suggested that disabled older adults and chronically ill older persons are more likely to report unmet needs [18]. However, few studies have analyzed the inequality in unmet healthcare needs among middle-aged and older adults. Hoebel et al. found that individuals with low socioeconomic status reported higher perceptions of unmet needs, and this association was observed for various types of health services [19]. Reeves et al. reported that greater public pension benefits play a crucial role in mitigating disparities in unmet healthcare needs among older individuals [20].

In China, several studies have been conducted on healthcare utilization and inequality among the middle-aged and older populations. Zhou et al. suggested that medical insurance plays a significant role in enhancing healthcare utilization [21]. Hu et al. reported that the healthcare needs of females, older adults, and older adults in rural areas were more likely to be reduced by their income [22]. Li et al. reported the existence of pro-rich inequity in healthcare utilization among middle-aged and older adults in China [23]. Fu et al. reported that middle-aged and older individuals with chronic non-communicable diseases in China face a pro-rich unfairness in inpatient services utilization [24]. Deng et al. suggested that pro-socioeconomically disadvantaged inequalities exist in access to community-based diabetes examination [25]. Fu et al. found the highest degree of inequality in preventive care, and it favored the rich, while the lowest degree was found for outpatient care [26]. Wu et al. discovered that the accessibility to health services for older adults remained pro-rich due to several factors such as household income, family size, and social medical insurance [27]. However, studies on unmet healthcare needs among Chinese middle-aged and older adults are limited. Li et al. found that middle-aged and older adults with one or multiple chronic diseases have a higher probability of reporting non-use of outpatient and inpatient services [28]. Gao et al. analyzed and reported the reasons for unmet healthcare needs among middle-aged and older adults, including financial difficulties, low acceptability, and inadequate health resources [29]. Kailembo et al. suggested that underutilization of oral health services among the least educated was almost 50% higher than that among the highest educated in China [30].

Several studies have focused on healthcare utilization and inequality among middle-aged and older adults, but little attention has been paid to the inequalities in unmet healthcare needs. To the best of our knowledge, no research has analyzed the inequality of unmet healthcare needs among middle-aged and older adults during the progression toward UHC in China. Exploring unmet healthcare needs and associated inequalities holds great significance for China in achieving UHC. In this study, we analyzed the prevalence and inequality of unmet healthcare needs among middle-aged and older adults in eastern China and identified its main contributors. We used data from the National Health Service Survey conducted in Jiangsu Province, China, between 2008 and 2018, a period coinciding with China’s progression toward UHC since NHCR.

## Methods

### Data sources

The data for this study were collected from the fourth, fifth, and sixth National Health Service Surveys (NHSSs) of Jiangsu Province in 2008, 2013, and 2018, respectively. These surveys covered the period of China’s progression toward UHC since the NHCR. The NHSS is carried out every five years by the National Bureau of Statistics, using a multi-stage stratified cluster sampling method. The surveys involved 31 provinces, 156 counties, 780 townships, and 1560 villages, with 60 households being selected in each sampled village. A total of 93,600 households were surveyed nationwide. Jiangsu Province is highly representative in terms of population, economic development level, and health resources allocation in the eastern China [31, 32]. The NHSS in Jiangsu Province sampled 19 counties, with 7021 respondents in 2008, 10,466 in 2013, and 11,550 in 2018, respectively. Therefore, the pooled cross-sectional data were used to analyze the unmet healthcare needs in this study. This type of data can provide valuable insights, including an increased sample size, temporal analysis, and diverse representation. However, researchers must carefully address potential flaws, such as measurement error and modeling complexities. The survey questionnaire systematically collects extensive data on demographic and socioeconomic attributes, healthcare utilization patterns, unmet healthcare needs, and other relevant factors pertaining to individuals residing in both urban and rural areas. As the target population of this study is middle-aged and older adults, participants younger than 45 years were excluded. After removing data with missing values, the final sample sizes for analysis were 3499 participants in 2008, 6658 in 2013, and 6281 in 2018, yielding a total of 16,438 participants.

### Data analysis

#### Measuring unmet healthcare needs

In this study, the unmet healthcare needs were categorized into non-use of outpatient services, non-use of inpatient services, and lack of access to physical examinations. The prevalence of non-use of outpatient services was determined by asking respondents the following questions: “Have you been ill for the last 14 days?” and “What was the main reason for not seeking outpatient treatment?” If the participant answered that he/she had been ill and did not seek outpatient treatment within the last 14 days, it was considered an unmet need for outpatient services. To estimate the prevalence of non-use of inpatient services, participants were asked the following question: “In the past year, did a doctor suggest that you needed inpatient care but you did not get hospitalized?” If the participant answered affirmatively to this question, it was classified as an unmet need for inpatient services. To determine the prevalence of non-use of physical examinations, participants were asked the following question: “In the past year, did you take the physical examination?” If a participant answered negatively to this question, it was regarded as an unmet need for physical examinations. Individuals should have at least one physical examination every year.

#### Regression analysis of factors associated with unmet healthcare needs

Here, we defined ${X}_{k}$ as the factors related to the prevalence of unmet healthcare needs. The study selected associated factors based on Andersen’s healthcare utilization model, including predisposing factors (demographics, social structure, health beliefs), enabling factors (access to healthcare, social insurance, economic status), and need factors (health status, chronic conditions, depression). The linear regression analysis equation for the prevalence of unmet healthcare needs was as follows:

1 $${Y_i} = \sum\limits_{} {_k{\beta _k}} {X_{ki}} + {\varepsilon _i}$$

where *Y* denotes the prevalence of unmet healthcare needs; ${X}_{k}$ are related factors, including predisposing factors (gender, age, marital status, employment status, education level, family sizes), enabling factors (distance to the nearest medical facility, area of residence, social medical insurance, economic status), and need factors (self-reported health, and chronic diseases and depression); and ${\varepsilon _i}$ is the error term. Logistic regression modelling was used to estimate the factors related to the prevalence of unmet healthcare needs, given that *Y* is a binary variable.

#### Measurement of concentration index of unmet healthcare needs

The concentration index (CI) was used to measure the degree of socioeconomic-related inequality in a health or healthcare variable. CI is defined as twice the area between the concentration curve and the line of equality, as proposed by Wagstaff et al. [33]. The concentration curve depicts shares of the health variable against quantiles of the living standards variable. In this paper, the inequality of unmet healthcare needs among middle-aged and older adults was examined with income tertiles, beginning with the poorest and ending with the richest:

2 $$CI = \frac{2}{\mu }COV(h,r)$$

where $h$ is the measure of actual health service underutilization, $\mu$ is its mean, and $r$ is the relative percentile rank of an individual in the distribution of economic status. Here, CI was defined as the distribution of non-use of outpatient services, inpatient services, or physical examinations across the population as ranked by per capita of household income. Individuals were sorted in ascending order of per capita of household income and grouped into three income tertiles. The range of CI was − 1 to 1. When the concentration curve was below the diagonal, the CI was positive, indicating that unmet healthcare needs favor the rich. A negative CI value indicated that the poor were more likely to have unmet healthcare needs. A CI value of zero indicated absolute fairness. Furthermore, the pro-rich pattern was more significant if the positive value was greater and the pro-poor pattern was more significant if the magnitude of negative values was greater.

#### Decomposition of CI of unmet healthcare needs

Using the decomposition method proposed by Wagstaff et al., factors that contributed to the inequality are assessed. The CI of unmet healthcare needs can be decomposed into individual factors that contribute to inequality, where each contribution is the product of the sensitivity of unmet healthcare needs to a given factor and the degree of inequality related to income for that factor. The decomposition analysis estimates how determining factors contribute proportionally to the inequality of unmet healthcare needs among middle-aged and older adults. The CI decomposition was calculated as follows:

3 $$CI = \sum\limits_{} {_k\left( {\frac{{{\beta _k}}}{{{{\mathop X\limits^ - }_k}}}} \right)} C{I_k} + \frac{{GC{I_\varepsilon }}}{{\mathop Y\limits^ - }}$$

4 $${\text{contribution}}\,{\text{rate}} = \frac{CI_{k}}{CI}$$

where *Y* is the mean of the prevalence of unmet healthcare needs; ${\stackrel{-}{X}}_{k}$ is the mean of ${X}_{k}$; ${CI}_{k}$ is the concentration index of ${X}_{k}$; and $GC{I_\varepsilon }$ is the generalized concentration index for the error term $\varepsilon$.

All analyses were performed using Stata version 17. Two-sided *p*-values less than 0.1 denoted statistical significance.

## Results

### Sample characteristics

As shown in Table 1, the total sample comprised 16,438 respondents older than 45 years old in Jiangsu Province. Of all respondents, 45.1% (7418) were urban residents and 54.9% (9020) were rural residents. The NHSS in 2008, 2013, and 2018 accounted for 21.3%, 40.5%, and 38.2% of the sample, respectively. Nearly four-fifths of middle-aged and older adults (78.9%) reported living no farther than 2 km from a medical facility. More than two-thirds (73.6%) resided in families comprising three or more members. More participants in the highest income group resided in urban areas (51.5%) than in rural areas (20.6%). The rate of coverage of social medical insurance surpassed 98% for both areas. The proportion of city dwellers with one or multiple chronic diseases (35.6% and 21.9%) exceeded that of rural residents (28.1% and 13.5%). More than 60% of respondents from both areas described their self-reported health status as excellent.

**Table 1:** Sample characteristics stratified by area of residence (urban and rural)

Variables	Total, *n* = 16,438	Urban, *n* = 7418 (45.1%)	Rural, *n* = 9020 (54.9%)	*P*-value
Year				0.000
2008	21.30%	12.80%	28.30%	
2013	40.50%	47.60%	34.70%	
2018	38.20%	39.60%	37%	
Predisposing factors				
Sex				0.190
Male	48.60%	48%	49.10%	
Female	51.40%	52%	50.90%	
Age (years)				0.000
45–59	48.90%	41.50%	54.90%	
60–74	38.70%	43.80%	34.60%	
≥ 75	12.40%	14.70%	10.50%	
Marital status				0.399
Married	87.20%	87%	87.40%	
Not married	12.80%	13%	12.60%	
Employment status				0.000
Employed	54.60%	29.90%	74.90%	
Retired	19.30%	17.50%	20.70%	
Not employed	26.20%	52.60%	4.40%	
Education level				0.000
Lower secondary and below	81.10%	70.40%	89.90%	
Upper secondary and vocational training	15.10%	22.30%	9.30%	
Tertiary	3.80%	7.30%	0.90%	
Family size				0.000
≤ 2	26.40%	30.10%	23.40%	
≥ 3	73.60%	69.90%	76.60%	
Enabling factors				
Distance to the nearest medical facility				0.000
≤ 2 km	78.90%	80.30%	77.80%	
> 2 km	21.10%	19.70%	22.20%	
Social health insurance				0.253
Yes	98.20%	98.10%	98.30%	
No	1.80%	1.90%	1.70%	
Income level				0.000
Tertile 1 (lowest, 10,440 yuan and below)	33.40%	17.90%	46.10%	
Tertile 2 (10,440 to 19,600 yuan)	32%	30.50%	33.20%	
Tertile 3 (highest, 19,600 yuan and above)	34.60%	51.50%	20.60%	
Need factors				
Self-reported health				0.663
Excellent	64.30%	63.70%	64.70%	
Good	18.40%	19.60%	17.40%	
Fair	15.10%	14.80%	15.40%	
Poor	2.30%	2%	2.50%	
Chronic diseases				0.000
0	51.20%	42.50%	58.40%	
1	31.50%	35.60%	28.10%	
≥ 2	17.30%	21.90%	13.50%	
Depression				0.000
Not depressed	92.60%	95.30%	90.40%	
Depressed	7.40%	4.70%	9.60%	

### Unmet healthcare needs among middle-aged and older adults from 2008 to 2018

The findings presented in Table 2 demonstrate that the prevalence of non-use of outpatient, inpatient services and physical examinations among the entire sampled middle-aged and older adults were 12.86%, 2.22%, and 48.89%, respectively. The rate of non-use of outpatient services increased over time (2008: 10.95%; 2013: 12.03%; and 2018: 14.81%), while that of unmet inpatient services was lower and was maintained across all the years examined (2008: 2.46%; 2013: 1.92%; and 2018: 2.40%). The rate of non-use of physical examinations demonstrated a marked decline from 2008 to 2013 (2008: 70.65%; 2013: 41.71%) but slightly increased in 2018 (44.37%). Compared to their rural counterparts, urban residents had a lower probability of non-use of inpatient services and physical examinations, with the most significant disparity in non-use of physical examinations (urban: 39.39%; rural: 56.70%). However, the rate of non-use of outpatient services similarly increased for both urban and rural areas from 2008 to 2018 (urban: 10.89–16.05%; rural: 10.97–13.71%). The rate of non-use of physical examinations significantly decreased from 2008 to 2013 among rural middle-aged and older adults (2008: 79%; 2013: 44.47%), albeit with a slight increase in 2018 (51.09%). However, urban areas witnessed a continuous decline from 2008 to 2018 (2008: 48.10%; 2013: 39.27%; 2018: 36.73%).

**Table 2:** Prevalence of unmet healthcare needs among middle-aged and older adults

	Total	Urban	Rural
Non-use of outpatient services	2114 (12.86%)	1016 (13.70%)	1098 (12.17%)
2008	383 (10.95%)	103 (10.89%)	280 (10.97%)
2013	801 (12.03%)	441 (12.49%)	360 (11.52%)
2018	930 (14.81%)	472 (16.05%)	458 (13.71%)
Non-use of inpatient services	365 (2.22%)	138 (1.86%)	227 (2.52%)
2008	86 (2.46%)	23 (2.43%)	63 (2.47%)
2013	128 (1.92%)	56 (1.59%)	72 (2.30%)
2018	151 (2.40%)	59 (2.01%)	92 (2.75%)
Non-use of physical examinations	8036 (48.89%)	2922 (39.39%)	5114 (56.70%)
2008	2472 (70.65%)	455 (48.10%)	2017 (79%)
2013	2777 (41.71%)	1387 (39.27%)	1390 (44.47%)
2018	2787 (44.37%)	1080 (36.73%)	1707 (51.09%)

### Factors associated with unmet healthcare needs among middle-aged and older adults

The findings in Table 3 suggest that some factors are significantly associated with unmet healthcare needs among middle-aged and older adults. Females were significantly less likely to report non-use of inpatient services (OR = 0.736) and physical examinations (OR = 0.789). Moreover, individuals aged 60 years and above were less likely to report non-use of physical examinations (OR = 0.634, 0.791). Unmarried individuals were less likely to report non-use of outpatient services (OR = 0.800); however, the opposite was true for non-use of physical examinations (OR = 1.222). Additionally, middle-aged and older adults who were not employed demonstrated a higher probability of non-use of outpatient services (OR = 1.349), while those who received higher education had a lower risk of unmet needs for physical examination (OR = 0.773, 0.450). Individuals living in larger families were less likely to report non-use of both outpatient (OR = 0.864) and inpatient services (OR = 0.818) but demonstrated a higher probability of unmet needs for physical examination (OR = 1.184).

**Table 3:** Logistic regression of unmet healthcare needs among middle-aged and older adults

Variables	Non-use of outpatient services		Non-use of inpatient services		Non-use of physical examinations	
	OR	95% confidence interval	OR	95% confidence interval	OR	95% confidence interval
Year, ref.: 2008						
2013	1.199**	(1.031, 1.394)	0.826	(0.614, 1.112)	0.315***	(0.287, 0.346)
2018	1.165**	(1.006, 1.350)	0.916	(0.687, 1.221)	0.349***	(0.318, 0.384)
**Predisposing factors**						
Sex, ref.: male	0.936	(0.841, 1.041)	0.736***	(0.592, 0.916)	0.789***	(0.737, 0.845)
Age, ref.: 45–59 years						
60–74 years	0.889*	(0.781, 1.011)	0.931	(0.711, 1.220)	0.634***	(0.584, 0.687)
≥ 75 years	0.936	(0.780, 1.122)	1.039	(0.722, 1.493)	0.791***	(0.697, 0.898)
Marital status, ref.: married	0.800***	(0.682, 0.938)	0.815	(0.591, 1.124)	1.222***	(1.098, 1.360)
Employment status, ref.: employed						
Retired	0.914	(0.783, 1.067)	0.793	(0.588, 1.069)	0.836***	(0.758, 0.922)
Not employed	1.349***	(1.141, 1.596)	0.835	(0.580, 1.201)	0.607***	(0.545, 0.676)
Education level, ref.: lower secondary and below						
Upper secondary and vocational training	0.851**	(0.726, 0.997)	0.865	(0.605, 1.237)	0.773***	(0.701, 0.852)
Tertiary	0.850	(0.642, 1.124)	0.230**	(0.072, 0.734)	0.450***	(0.370, 0.546)
Family size, ref.: ≤2	0.864**	(0.769, 0.970)	0.818*	(0.645, 1.037)	1.184***	(1.094, 1.281)
**Enabling factors**						
Distance to the nearest medical facility, ref.: ≤2 km	0.896*	(0.786, 1.020)	1.202	(0.935, 1.545)	1.094**	(1.010, 1.185)
Social health insurance, ref.: yes	1.655**	(1.122, 2.442)	1.930**	(1.038, 3.588)	1.539***	(1.175, 2.015)
Area of residence, ref.: urban	1.139*	(0.993, 1.307)	1.167	(0.878, 1.550)	1.036	(0.954, 1.125)
Income level, ref.: Tertile 1						
Tertile 2	0.848**	(0.741, 0.972)	0.753**	(0.573, 0.989)	0.890***	(0.819, 0.967)
Tertile 3	0.973	(0.844, 1.121)	0.828	(0.616, 1.112)	0.735***	(0.672, 0.804)
**Need factors**						
Self-reported health, ref.: excellent						
Good	1.158**	(1.013, 1.322)	1.846***	(1.362, 2.501)	1.018	(0.931, 1.114)
Fair	1.441***	(1.253, 1.658)	2.713***	(2.014, 3.654)	1.092*	(0.984, 1.211)
Poor	1.853***	(1.417, 2.424)	3.942***	(2.519, 6.169)	1.526***	(1.206, 1.930)
Chronic diseases, ref.: 0						
1	0.260***	(0.224, 0.302)	2.513***	(1.843, 3.425)	0.685***	(0.634, 0.739)
≥ 2	0.252***	(0.213, 0.298)	5.021***	(3.637, 6.931)	0.566***	(0.512, 0.626)
Depression, ref.: not depressed	1.147	(0.969, 1.357)	2.155***	(1.634, 2.841)	1.284***	(1.122, 1.471)

*P < 0.1, **P < 0.05, ***P < 0.01

Regarding enabling factors, residing more than 2 km away from the nearest medical facility was significantly correlated with a lower probability of non-use of outpatient services (OR = 0.896). Rural residents were more likely to report non-use of outpatient services than urban residents (OR = 1.139). Middle-aged and older adults without social medical insurance demonstrated a significantly higher probability of unmet needs for three types of healthcare services (OR = 1.655, 1.930, 1.539). In addition, individuals with middle-level income were less likely to have unmet healthcare needs (OR = 0.848, 0.753, 0.890) than those with low-level income. Individuals who self-reported their health status as poor or fair showed a higher likelihood of non-use of both outpatient and inpatient services. Middle-aged and older adults with one or multiple chronic diseases were less likely to report non-use of outpatient services (OR = 0.260, 0.252) and physical examinations (OR = 0.685, 0.566), but were more likely to report unmet needs for hospitalization (OR = 2.513, 5.021). Those who suffered from depression showed a higher likelihood of non-use of inpatient services (OR = 2.155) and physical examinations (OR = 1.284).

### Inequality of unmet healthcare needs among middle-aged and older adults

Table 4 shows the CIs of unmet healthcare needs among middle-aged and older adults in China. The total CIs for non-use of outpatient, inpatient services and physical examinations were − 0.019, − 0.159, and − 0.114, respectively. This indicated that unmet healthcare needs were disproportionately predominant among the poor. The pro-poor inequalities for three types of unmet healthcare needs were more pronounced among rural middle-aged and older adults (− 0.062, − 0.199, − 0.091) than among their urban counterparts (0.060, − 0.034, − 0.065). Examining the trend in changes, the absolute values of the CIs of non-use of outpatient and hospitalization underutilization decreased from 2008 to 2018, suggesting that the pro-poor inequality in unmet healthcare needs was gradually alleviated. The absolute values of the CIs of non-use of physical examinations significantly decreased from 2008 to 2013 (2008: −0.130; 2013: −0.033) but slightly increased from 2013 to 2018 (2013: −0.033; 2018: −0.060). In urban areas, the unmet needs for outpatient services demonstrated a pro-rich inequality from 2008 to 2018 with positive CIs (2008: 0.045; 2013: 0.057; 2018: 0.053). Conversely, the non-use of outpatient services consistently demonstrated inequality, being predominant among the poor during the same period in rural areas (2008: −0.053; 2013: −0.075; 2018: −0.058). Regarding unmet needs for hospitalization, pro-poor inequality showed a marked decrease from 2008 to 2018 in urban areas (2008: −0.308, 2018: −0.045); however, the pro-poor inequality in rural areas persisted and was relatively stable (2008: −0.243, 2018: −0.193). Finally, pro-poor inequality in non-use of physical examinations was alleviated in both urban and rural areas from 2008 to 2018 (urban: 2008: −0.104, 2018: −0.030; rural: 2008: −0.075, 2018: −0.028).

**Table 4:** Concentration indexes of unmet healthcare needs among middle-aged and older adults

	Total	Urban	Rural
Non-use of outpatient services	−0.019	0.060	−0.062
2008	−0.068	0.045	−0.053
2013	−0.026	0.057	−0.075
2018	0.001	0.053	−0.058
Non-use of inpatient services	−0.159	−0.034	−0.199
2008	−0.219	−0.308	−0.243
2013	−0.153	0.043	−0.273
2018	−0.149	−0.045	−0.193
Non-use of physical examinations	−0.114	−0.065	−0.091
2008	−0.130	−0.104	−0.075
2013	−0.033	−0.058	0.015
2018	−0.060	−0.030	−0.028

### Decomposition of the inequality of unmet healthcare needs among middle-aged and older adults

We decomposed the total CIs of the three types of unmet healthcare needs based on the regression results. Table 5 presents the contributors to the pro-poor inequalities of unmet healthcare needs among middle-aged and older adults. Our findings showed that the majority of the observed pro-poor inequalities in the non-use of outpatient services can be attributed to living in rural areas (39.45%), self-reported health status (38.72%), having one or multiple chronic diseases (33.10%, 29.03%), and high income (27.75%). In contrast, being retired (− 19.95%) and not married (− 11.16%) negatively contributed to the inequalities. High income (25.53%), depression (17.91%), and urban or rural residence (14.18%) were the major positive contributors to the pro-poor inequalities in the non-use of inpatient services while having one or multiple chronic diseases (− 9.93%, − 3.47%) and being retired (− 3.85%) had negative contributions. The main positive contributions to the pro-poor inequalities in non-use of physical examinations were high income (18.42%), unemployment (14.77%), and urban or rural residence (12.59%). Retirement (− 2.77%), age (60–74: −0.68%; ≥75: −0.61%), and household counts of ≥ 3 (− 0.15%) had slightly negative contributions to the inequalities.

**Table 5:** Decomposition of the concentration indexes of unmet healthcare needs

	Non-use of outpatient services				Non-use of inpatient services				Non-use of physical examinations		
	Elasticity	CI	Contribution to CI (%)		Elasticity	CI	Contribution to CI (%)		Elasticity	CI	Contribution to CI (%)
Sex, ref.: male	−0.026	−0.025	−3.48%		−0.145	−0.001	−0.03%		−0.062	−0.001	−0.02%
Age, ref.: 45–59 years											
60–74 years	−0.035	0.001	0.12%		−0.038	−0.008	−0.18%		−0.100	−0.008	−0.68%
≥ 75 years	−0.007	−0.015	−0.59%		−0.001	−0.038	−0.03%		−0.018	−0.038	−0.61%
Marital status, ref.: married	−0.024	−0.087	−11.16%		−0.035	−0.086	−1.89%		0.017	−0.086	1.28%
Employment status, ref.: employed											
Retired	−0.015	−0.245	−19.95%		−0.030	−0.205	−3.85%		−0.015	−0.205	−2.77%
Not employed	0.070	0.304	−113.85%		−0.035	0.335	7.37%		−0.050	0.335	14.77%
Education level, ref.: lower secondary and below											
Upper secondary and vocational training	−0.015	0.289	23.51%		−0.014	0.260	2.33%		−0.020	0.260	4.58%
Tertiary	−0.005	0.557	14.19%		−0.026	0.573	9.35%		−0.013	0.573	6.37%
Family size, ref.: ≤2	−0.069	−0.001	−0.39%		−0.177	0.006	0.63%		0.030	0.006	−0.15%
Distance to the nearest medical facility, ref.: ≤2 km	−0.016	−0.074	−6.12%		0.038	−0.045	1.06%		0.007	−0.045	0.26%
Social health insurance, ref.: yes	0.006	−0.287	8.53%		0.014	−0.260	2.29%		0.006	−0.260	1.37%
Area of residence, ref.: urban	0.032	−0.229	39.45%		0.110	−0.206	14.18%		0.070	−0.206	12.59%
Income level, ref.: Tertile 1											
Tertile 2	−0.036	−0.015	−2.86%		−0.093	0.011	0.65%		−0.013	0.011	0.13%
Tertile 3	−0.010	0.544	27.75%		−0.072	0.565	25.53%		−0.037	0.565	18.42%
Self-reported health, ref.: excellent											
Good	0.022	−0.072	8.64%		0.071	−0.072	3.18%		0.005	−0.072	0.33%
Fair	0.060	−0.122	38.72%		0.172	−0.107	11.53%		0.008	−0.107	0.75%
Poor	0.019	−0.233	23.87%		0.062	−0.230	8.98%		0.005	−0.230	0.94%
Chronic diseases, ref.: 0											
1	−0.549	0.011	33.10%		0.168	0.033	−3.47%		−0.066	0.033	1.92%
≥ 2	−0.304	0.018	29.03%		0.298	0.053	−9.93%		−0.053	0.053	2.49%
Depression, ref.: not depressed	0.012	−0.248	15.59%		0.139	−0.205	17.91%		0.010	−0.205	1.89%

## Discussion

Achieving UHC presents a fundamental challenge in addressing unmet healthcare needs and inequalities among middle-aged and older adults, especially with the rapid population aging in China. Several scholars have researched healthcare utilization and inequality among middle-aged and older adults, but little attention has been paid to the inequalities in unmet healthcare needs. To our knowledge, this is the first study to measure the inequality of unmet healthcare needs among middle-aged and older adults during China’s progression toward UHC.

### Prevalence and trends of unmet healthcare needs

In this study, we found that 12.86%, 2.22%, and 48.89% of the entire sample of middle-aged and older adults reported non-use of outpatient, inpatient services and physical examinations, respectively. The high rate of unmet needs for physical examination may be attributed to the lack of health awareness among middle-aged and older adults or non-coverage of physical examination items by social medical insurance schemes, which prevents access to preventive care services. Hence, addressing these challenges will require targeted interventions that promote health education and awareness and policy reforms that expand the coverage of social health insurance schemes for preventive care services. The prevalence of non-use of inpatient services was relatively low, which is consistent with previous research studies [28, 29, 34]. Individuals are more likely to utilize inpatient services when they perceive an increase in disease severity or acuity [35].

The prevalence of non-use of outpatient services increased from 10.95% in 2008 to 14.81% in 2018. The coverage of social medical insurance in China has been significantly expanding since NHCR and is approaching universal coverage. However, the increase in the prevalence of unmet needs for outpatient health services among middle-aged and older adults may indicate the persistence of various barriers to access to outpatient services. However, with the improvement of living conditions in China, the unmet need for physical examinations markedly decreased from the initial 70.65% in 2008 to 44.37% in 2018. In addition, middle-aged and older adults in rural areas demonstrated a higher prevalence of unmet needs for inpatient services and physical examinations than their urban counterparts, which is consistent with the reports of a previous study [36]. This may be attributed to the gaps in economic and social development and the allocation of health resources between urban and rural areas.

### Factors associated with unmet healthcare needs

Unmet needs for outpatient and physical examinations decreased with increasing age. This may reflect changes in health-seeking behavior and care utilization patterns over the life course. The older a person gets, the more likely their physical condition will deteriorate, and their unmet healthcare needs may decrease [9]. Contrary to the reports of some studies [37–40], middle-aged and older women demonstrated a lower prevalence of unmet healthcare needs than men. This may be because women are more likely to adhere to medical advice and treatment regimens than men, which helps to reduce rates of non-use of health services [41, 42]. We also found that individuals with higher levels of education are significantly less likely to have unmet healthcare needs, similar to the reports of previous studies [43, 44]. Individuals with higher levels of education are more likely to have more knowledge about available healthcare services, which facilitates better access to them. Higher income was also associated with a lower prevalence of unmet healthcare needs, as reported previously reports [45, 46]. Middle-aged and older adults with social medical insurance demonstrated a lower probability of unmet healthcare needs, similar to previous reports [47, 48]. Individuals with depression were significantly more likely to have unmet healthcare needs, as previously reported [16, 49, 50]. This finding underscored the potential negative impact of mental health conditions on access to necessary healthcare services. Respondents with one or multiple chronic diseases were less likely to have unmet needs for outpatient and physical examination services but more likely to not use hospitalization services. The possible reason is that individuals with chronic diseases may have more frequent healthcare visits for the management of their conditions, thereby reducing the probability of unmet needs for outpatient and physical examination services. However, the unmet need for hospitalization services may be due to barriers related to affordability, accessibility, or other factors.

### Inequality of unmet healthcare needs and its decomposition

Overall, the unmet healthcare needs showed pro-poor inequality, indicating that middle-aged and older adults with lower socioeconomic status are more likely to have unmet healthcare needs, as previously reported [19, 29]. Our research findings indicate that the pro-poor inequality of unmet outpatient service needs among middle-aged and older adults was declining between 2008 and 2018. However, urban residents demonstrated a pro-rich inequality from 2008 to 2018. A possible explanation is that urban dwellers with higher socioeconomic status tend to have higher expectations for healthcare services compared to rural individuals. Nonetheless, the current outpatient service system in urban China faces issues such as overcrowded medical resources, long waiting times, and lower quality of care, leading to unmet outpatient service needs for urban middle-aged and elderly populations. In contrast, rural residents demonstrated a stable pro-poor inequality, indicating that rural middle-aged and older residents with low income were more likely to not use outpatient services. The degree of pro-poor inequality was not very high; however, targeted interventions and policies were still needed to address the challenges faced by vulnerable middle-aged and older adults residing in rural areas. Factors, such as living in rural areas, self-reported health status, having one or multiple chronic diseases, and high income are major positive contributors to the pro-poor inequality of unmet outpatient services needs. Hence, policies aimed at reducing these inequalities should address these socioeconomic factors, such as implementing strategies that narrow the income gap, optimize urban and rural health resources, and strengthen the prevention and control of chronic diseases.

There was significant pro-poor inequality of unmet inpatient service needs; middle-aged and older adults with low income are more likely to have unmet needs for inpatient services. We found that the pro-poor inequality substantially declined during the progression toward UHC in urban areas; however, the pro-poor inequality persisted in rural areas. Low income, poor health resources, and low quality of healthcare available in rural areas may have contributed to this disparity. Therefore, strategies for narrowing the income gap between urban and rural areas and improving the allocation and quality of healthcare resources in rural areas may be practical for alleviating the pronounced pro-poor inequality of unmet inpatient services need. Major positive contributors to the inequality of unmet inpatient services needs were high income, depression, and living in rural areas. High income emerged as the primary contributor to the inequality. This underscores the significance of income as a factor underlying unequal access to healthcare. Financial considerations may hinder individuals from seeking necessary hospital care, resulting in unmet inpatient needs and exacerbation of the inequality. Thus, policies designed to address these inequalities should prioritize the economic risks of diseases among middle-aged and older populations. Finally, the unmet need for physical examination services also demonstrated a pro-poor inequality among middle-aged and older adults. Compared with 2008, it significantly decreased in 2013 and 2018, indicating progress in addressing suboptimal access to preventive healthcare services and inequity among middle-aged and older adults since NHCR in China. High income, unemployment, and living in rural areas positively contributed to the observed inequality.

This study innovatively adopts UHC as its research perspective, using data from 2008 to 2018, covering China’s progression toward UHC since the NHCR. It focuses on middle-aged and older adults, a crucial segment given China’s aging population. Considering the significant advancements in UHC in Eastern China [31, 51, 52], these findings provide invaluable insights for the development of healthcare systems in other regions of China and other developing countries. Some limitations of our study must be acknowledged. Firstly, our study only considered individuals who perceived a need for healthcare but did not receive it. Consequently, the prevalence of unmet healthcare needs may have been underestimated to some extent since we did not take into account those who did not perceive the need. Secondly, pooled cross-sectional data used in this study did not allow for causal conclusions.

## Conclusion

Our findings revealed that the prevalence of unmet needs for outpatient services among middle-aged and older adults in eastern China increased from 2008 to 2018. In contrast, the prevalence of unmet needs for inpatient services was maintained low over the same duration. Notably, the unmet needs for physical examinations among middle-aged and older adults demonstrated a remarkable decrease from 2008. Individuals residing in rural areas exhibited a higher prevalence of unmet needs for inpatient services and physical examinations than their urban counterparts. The unmet healthcare needs were disproportionately predominant among the poor. The pro-poor inequalities of the unmet healthcare needs were mitigated during the progression toward UHC; however, those for outpatient and inpatient services persist among middle-aged and older adults residing in rural areas. Several socioeconomic factors have been associated with unmet healthcare needs and related inequalities. Therefore, policy interventions aimed at reducing unmet healthcare needs and associated inequalities should address the socioeconomic determinants of healthcare utilization among middle-aged and older adults.

## Data Availability

The datasets used in the current study are not publicly available due to the confidentiality policy but are available from the corresponding author on reasonable request.

## Abbreviations

UHC: universal health coverage
NHSS: National Health Service Survey
CI: concentration index
SDG: Sustainable Development Goal
NHCR: New Health Care Reform

## References

[1] Kutzin J (2013). Health financing for universal coverage and health system performance: concepts and implications for policy. Bull World Health Organ.

[2] Travis P, Bennett S, Haines A, Pang T, Bhutta Z, Hyder AA, Pielemeier NR, Mills A, Evans T (2004). Overcoming health-systems constraints to achieve the Millennium Development Goals. Lancet.

[3] Ozturk S, Basar D (2020). Equity in utilization of health care services in Turkey: an index based analysis. East Mediterr Health J.

[4] Sun J, Wang Y, Zhang Y, Li L, Li H, Liu T, Zhang L. Research on the risk governance of fraudulent reimbursement of patient consultation fees. Front Public Health 2024, 12. 10.3389/fpubh.2024.1339177 PMC10895054 38410668

[5] Zhou S, Zhou C, Yuan Q, Wang Z. Universal Health Insurance Coverage and the Economic Burden of Disease in Eastern China: a Pooled Cross-sectional Analysis from the National Health Service Survey in Jiangsu Province. Front Public Health 2022, 10. 10.3389/fpubh.2022.738146 PMC8858842 35198521

[6] Li A, Shi Y, Yang X, Wang Z. Effect of Critical Illness Insurance on Household Catastrophic Health expenditure: the latest evidence from the National Health Service Survey in China. Int J Environ Res Public Health 2019, 16(24). 10.3390/ijerph16245086 PMC6950570 31847072

[7] Patenaude B, Rao KD, Peters DH (2021). An empirical examination of the inequality of Forgone Care in India. Health Syst Reform.

[8] Lucevic A, Pentek M, Kringos D, Klazinga N, Gulacsi L, Brito Fernandes O, Boncz I, Baji P (2019). Unmet medical needs in ambulatory care in Hungary: forgone visits and medications from a representative population survey. Eur J Health Econ.

[9] Rottger J, Blumel M, Koppen J, Busse R (2016). Forgone care among chronically ill patients in Germany-results from a cross-sectional survey with 15,565 individuals. Health Policy.

[10] Zhou C, Ji C, Chu J, Medina A, Li C, Jiang S, Zheng W, Liu J, Rozelle S (2015). Non-use of health care service among empty-nest elderly in Shandong, China: a cross-sectional study. BMC Health Serv Res.

[11] Chen J, Hou F (2002). Unmet needs for health care. Health Rep.

[12] Vahedi S, Yazdi-Feyzabadi V, Amini-Rarani M, Mohammadbeigi A, Khosravi A, Rezapour A (2020). Tracking socio-economic inequalities in healthcare utilization in Iran: a repeated cross-sectional analysis. BMC Public Health.

[13] Johar M, Soewondo P, Pujisubekti R, Satrio HK, Adji A (2018). Inequality in access to health care, health insurance and the role of supply factors. Soc Sci Med.

[14] Somkotra T, Detsomboonrat P (2009). Is there equity in oral healthcare utilization: experience after achieving Universal Coverage. Community Dent Oral Epidemiol.

[15] Joe W, Rudra S, Subramanian SV (2015). Horizontal inequity in Elderly Health Care utilization: evidence from India. J Korean Med Sci.

[16] Mielck A, Kiess R, von dem Knesebeck O, Stirbu I, Kunst AE (2009). Association between forgone care and household income among the elderly in five western European countries - analyses based on survey data from the SHARE-study. BMC Health Serv Res.

[17] Eimontas J, Gegieckaite G, Zamalijeva O, Pakalniskiene V. Unmet Healthcare needs Predict Depression symptoms among older adults. Int J Environ Res Public Health 2022, 19(15). 10.3390/ijerph19158892 PMC9330083 35897261

[18] Momtaz YA, Hamid TA, Ibrahim R (2012). Unmet needs among disabled elderly malaysians. Soc Sci Med.

[19] Hoebel J, Rommel A, Schroder SL, Fuchs J, Nowossadeck E, Lampert T. Socioeconomic inequalities in Health and Perceived Unmet needs for Healthcare among the Elderly in Germany. Int J Environ Res Public Health 2017, 14(10). 10.3390/ijerph14101127 PMC5664628 28954436

[20] Reeves A, McKee M, Mackenbach J, Whitehead M, Stuckler D (2017). Public pensions and unmet medical need among older people: cross-national analysis of 16 European countries, 2004–2010. J Epidemiol Community Health.

[21] Zhou Y, Wushouer H, Vuillermin D, Ni B, Guan X, Shi L (2020). Medical insurance and healthcare utilization among the middle-aged and elderly in China: evidence from the China health and retirement longitudinal study 2011, 2013 and 2015. BMC Health Serv Res.

[22] Hu J, Huang C-C (2016). Health Service utilization and expenditure of the Elderly in China. Asian Soc Work Policy Rev.

[23] Li C, Dou L, Wang H, Jing S, Yin A. Horizontal inequity in Health Care utilization among the Middle-aged and Elderly in China. Int J Environ Res Public Health 2017, 14(8). 10.3390/ijerph14080842 PMC5580546 28933772

[24] Fu XZ, Wang LK, Sun CQ, Wang DD, He JJ, Tang QX, Zhou QY (2020). Inequity in inpatient services utilization: a longitudinal comparative analysis of middle-aged and elderly patients with the chronic non-communicable diseases in China. Int J Equity Health.

[25] Deng Q, Wei Y, Chen Y (2022). Inequalities in access to community-based diabetes examination and its impact on healthcare utilization among middle-aged and older adults with diabetes in China. Front Public Health.

[26] Fu L, Fang Y, Dong Y (2022). The healthcare inequality among middle-aged and older adults in China: a comparative analysis between the full samples and the homogeneous population. Health Econ Rev.

[27] Wu H, Liu Y (2020). Examining inequality in utilisation of health management services for the elderly in rural Henan China. BMC Health Serv Res.

[28] Li X, Chen M, Wang Z, Si L (2018). Forgone care among middle aged and elderly with chronic diseases in China: evidence from the China Health and Retirement Longitudinal Study Baseline Survey. BMJ Open.

[29] Gao Q, Prina M, Wu YT, Mayston R. Unmet healthcare needs among middle-aged and older adults in China. Age Ageing 2022, 51(1). 10.1093/ageing/afab235 34923586

[30] Kailembo A, Preet R, Stewart Williams J (2018). Socioeconomic inequality in self-reported unmet need for oral health services in adults aged 50 years and over in China, Ghana, and India. Int J Equity Health.

[31] Wan S, Wang M (2024). Population mobility: spatial spillover effect of government health expenditure in China. Glob Health Action.

[32] Liu T, Li J, Chen J, Yang S. Regional differences and Influencing Factors of Allocation Efficiency of Rural Public Health Resources in China. Healthc (Basel) 2020, 8(3). 10.3390/healthcare8030270 PMC7551190 32823864

[33] Wagstaff A, van Doorslaer E (2003). Catastrophe and impoverishment in paying for health care: with applications to Vietnam 1993–1998. Health Econ.

[34] Zhuoga C, Cuomu Z, Li S, Dou L, Li C, Dawa Z (2023). Income-related equity in inpatient care utilization and unmet needs between 2013 and 2018 in Tibet, China. Int J Equity Health.

[35] Traverso CE, Walt JG, Kelly SP, Hommer AH, Bron AM, Denis P, Nordmann JP, Renard JP, Bayer A, Grehn F (2005). Direct costs of glaucoma and severity of the disease: a multinational long term study of resource utilisation in Europe. Br J Ophthalmol.

[36] Liu X, Li N, Liu C, Ren X, Liu D, Gao B, Liu Y (2016). Urban-rural disparity in utilization of preventive care services in China. Med (Baltim).

[37] Bryant T, Leaver C, Dunn J (2009). Unmet healthcare need, gender, and health inequalities in Canada. Health Policy.

[38] Tadiri CP, Gisinger T, Kautzky-Willer A, Kublickiene K, Herrero MT, Norris CM, Raparelli V, Pilote L, Consortium G-F (2021). Determinants of perceived health and unmet healthcare needs in universal healthcare systems with high gender equality. BMC Public Health.

[39] Nelson CH, Park J (2006). The nature and correlates of unmet health care needs in Ontario, Canada. Soc Sci Med.

[40] Levesque J-F, Pineault R, Hamel M, Roberge D, Kapetanakis C, Simard B, Prud’homme A. Emerging organisational models of primary healthcare and unmet needs for care: insights from a population-based survey in Quebec province. *BMC Family Practice* 2012, 13(66). 10.1186/1471-2296-13-66 PMC3431245 22748060

[41] Belhabib G, Lahyani M, Mhiri A, Gloulou O, Sahli J, Chouchane N (2018). Evaluation of factors for therapeutic adherence in diabetic patients. Le Pharmacien Hospitalier et Clinicien.

[42] Abdel Aal A, Youssef G, El Faramawy A, El Remisy D, El Deeb H, El Aroussy W, Ibrahim MM (2021). Registry of the Egyptian specialized hypertension clinics: sex-related differences in clinical characteristics and hypertension management among low socioeconomic hypertensive patients. J Clin Hypertens (Greenwich).

[43] Junfang W, Biao Z, Weijun Z, Zhang S, Yinyin W, Chen K (2009). Perceived unmet need for hospitalization service among elderly Chinese people in Zhejiang Province. J Public Health (Oxf).

[44] Hwang J (2018). Understanding reasons for unmet health care needs in Korea: what are health policy implications?. BMC Health Serv Res.

[45] Jou J, Upchurch DM, Johnson PJ. Delay and nonreceipt of needed healthcare in U.S. adults with household family members with serious health needs. Fam Syst Health 2023. 10.1037/fsh0000787 37036678

[46] Roh HL, Kim SD. Unmet Healthcare needs among the Elderly Korean Population: before and during the COVID-19 pandemic. Systems 2023, 11(9).

[47] Pagan JA, Pauly MV (2006). Community-level uninsurance and the unmet medical needs of insured and uninsured adults. Health Serv Res.

[48] Mao W, Zhang Y, Xu L, Miao Z, Dong D, Tang S (2020). Does health insurance impact health service utilization among older adults in urban China? A nationwide cross-sectional study. BMC Health Serv Res.

[49] Hu C, Yu W, Lv Y, Chen H, Deng Q, Zhang L. Study on the Health Status and Health Service utilization of the Elderly of a remote and poor village in a Mountainous Area in Jinzhai, Anhui. Int J Environ Res Public Health 2017, 14(4). 10.3390/ijerph14040408 PMC5409609 28417937

[50] Geitona M, Zavras D, Kyriopoulos J (2007). Determinants of healthcare utilization in Greece: implications for decision-making. Eur J Gen Pract.

[51] Yu X, Zhang W (2020). All-cause mortality rate in China: do residents in economically developed regions have better health?. Int J Equity Health.

[52] Li Z, Yu Q, Lu X, Liu Y, Ji B (2021). Efficacy of radiofrequency ablation versus laparoscopic liver resection for hepatocellular carcinoma in China: a comprehensive meta-analysis. Wideochir Inne Tech Maloinwazyjne.

